# Impact of Graphene Work Function on the Electronic Structures at the Interface between Graphene and Organic Molecules

**DOI:** 10.3390/nano9081136

**Published:** 2019-08-07

**Authors:** Haitao Wang, Xiangdong Yang, Weidong Dou, Peng Wang, Quanlin Ye, Xuxin Yang, Baoxing Li, Hongying Mao

**Affiliations:** 1 Department of Physics, Hangzhou Normal University, Hangzhou 311121, China; 2 Laboratory of Low-dimensional Carbon Materials, Physics Department, Shaoxing University, Shaoxing 312000, China; 3 Department of Applied Physics, College of Electronic and Information Engineering, Shandong University of Science and Technology, Qingdao 266590, China

**Keywords:** graphene, self-assembled monolayers, work function, band bending

## Abstract

The impact of graphene work function (WF) on the electronic structure at the graphene/organic interface has been investigated. WF manipulation of graphene is realized using self-assembled monolayers (SAMs) with different end groups. With this method, the upper surface of the functionalized graphene remains intact, and thus precludes changes of molecular orientation and packing structures of subsequently deposited active materials. The WF of NH_2_-SAM functionalized graphene is ~3.90 eV. On the other hand, the WF of graphene increases to ~5.38 eV on F-SAM. By tuning the WF of graphene, an upward band bending is found at the ZnPc/graphene interface on F-SAM. At the interface between C_60_ and NH_2_-SAM modified graphene, a downward band bending is observed.

## 1. Introduction

Graphene is a promising alternative to transparent electrodes in organic electronic devices [1,2,3,4,5,6,7,8], and its electronic, optical, and thermal properties have been extensively studied in recent years. Although graphene-based organic electronic devices have been successfully fabricated by many groups, the devices’ performance has remained low compared to conventional devices in which indium tin oxide (ITO) was used [9,10,11,12]. In order to achieve enhanced device performance, the interfacial electronic structure at electrode/active materials must be optimized [13,14,15,16,17,18,19,20,21,22]. For example, when the work function (WF) of the cathode is lower than the electron affinity (EA) of electron transport materials, the electron injection or collection at the cathode is improved [23]. On the other hand, anode materials with high WF are highly desirable for organic light emitting diodes (OLEDs) [24].

The insertion of an interfacial layer is one of the most commonly used surface functionalization methods to manipulate the WF of electrodes. Poly (3,4-ethylenedioxythiophene):poly (styrenesulfonate) (PEDOT:PSS) facilitated the extraction of holes at the active layer/anode interface [25]. Moreover, because of its deep-lying electronic state, MoO_3_ has been utilized for hole injection enhancement in related devices [26,27,28]. In other cases, surface modification with low WF interfacial layers [29,30,31], including LiF and CsCO_3_, was employed to decrease the WF of the electrode for better electron injection. In addition to the insertion of an interfacial layer, the manipulation of the graphene WF can also be realized using plasma treatments. O_2_ or SF_6_ plasma treatments have been used to increase the WF of graphene, which was attributed to the presence of oxygenated or fluorinated species on the graphene basal plane [32,33,34]. Although the effective manipulation of the graphene WF was realized with the interfacial layer or plasma treatments, the molecular orientation and packing structures of subsequently deposited active materials were inevitably disturbed. Our previous studies revealed that ClAlPc molecules adsorbed on graphene with their molecular π–plane nearly parallel to the graphene basal plane. However, for the growth of ClAlPc on bare ITO without graphene modification, the molecular orientation is random [35]. It is worth noting that changes of molecular orientation and packing structure have influenced the interfacial electronic structure, and orientation-dependent molecular electronic structures have been recently reported [36,37,38,39,40]. For surface modification with interfacial layers or plasma treatments, there are two factors that can have impacts on the interfacial energy levels. The first is the WF change of graphene after functionalization, and the second is the change of molecular orientation and packing structure. For better understanding the impact of the graphene WF on the electronic structure at the graphene/active material interface, a novel interfacial engineering approach has to be developed which separates the impact of the graphene WF change from that of molecular orientation and packing structure changes.

Recently, n-type and p-type doping of graphene using self-assembled monolayers (SAMs) with different end groups has been demonstrated [41,42]. In general, n-type doping led to an upward shift in the graphene Fermi level, while a downward shift of the graphene Fermi level was induced by p-type doping. This indicated that the WF tuning of graphene can be realized using SAMs with different end groups. More importantly, SAMs were constructed on SiO_2_ substrates. After the formation of SAMs, graphene was transferred on the top of SAMs, leaving the upper surface of SAM functionalized graphene intact as pristine graphene. Without the insertion of an interfacial layer or plasma treatments, the molecular orientation and packing structures of subsequently deposited active materials remained unchanged, which helped to separate the impact of the graphene WF from molecular orientation and packing structure changes. Using SAM modified graphene, we have successfully tuned the Fermi level of graphene without disturbing molecular orientations and packing structures of active materials on top. Model systems of graphene/organic molecule interfaces have been constructed, which simplifies factors affecting the interface energy levels compared with previous works.

In the present study, we demonstrated the manipulation of the graphene WF using different SAMs. As revealed by ultraviolet photoelectron spectroscopy (UPS) results, the Fermi level of graphene shifted upward on NH_2_-SAM, and the WF decreased to ~3.90 eV; in the case of graphene on F-SAM, its Fermi level moved downward, and the graphene WF increased to ~5.38 eV. The influence of the graphene WF on the interfacial energy levels was been investigated. An upward band bending was observed at the interface between ZnPc and F-SAM functionalized graphene, while a downward band bending was found for the growth of C_60_ on NH_2_-SAM functionalized graphene.

## 2. Materials and Methods 

### 2.1. Preparation of NH_2_-SAM and F-SAM on SiO_2_

The SiO_2_ substrate was treated with O_2_ plasma for 15 min to generate a hydrophilic surface for the formation of NH_2_-SAM and F-SAM (their molecular structures are shown in Figure S1). After O_2_ plasma treatments, SiO_2_ substrates were treated with piranha solution for 20 min, and then washed using deionized (DI) water. Finally, they were dried by nitrogen flow. In the case of NH_2_-SAM, 0.8 mL 3-aminopropyltriethoxysilane (33% solution in toluene by volume, Sigma-Aldrich) was added into a sealed vial together with clean SiO_2_ substrates under nitrogen environments. The vial was then heated to 80 °C and kept for 2 h before being thoroughly washed with toluene, methanol (HPLC grade, Fisher Scientific) and DI water. The functionalized SiO_2_ wafer was then immerged in DI water for more than 12 h at room temperature (RT), which is helpful to complete the hydrolysis of residual ethoxy groups. Finally, NH_2_-SAM functionalized SiO_2_ substrates were dried with nitrogen flow. For the formation of F-SAM, clean SiO_2_ substrates were placed in a sealed vial filled with 0.5 mL 1H, 1H, 2H, 2H-perfluorooctyltriethoxysilane (Sigma-Aldrich). The sealed vial was then heated in an oven at 120 °C for 1 h. After, F-SAM functionalized SiO_2_ wafers were rinsed by toluene, methanol, and DI water to remove the physically adsorbed F-SAM precursor molecules. Finally, they were dried by nitrogen gas. 

### 2.2. Graphene Growth and Transfer

The growth of graphene on copper foil was in a custom designed low-pressure chemical vapor deposition (LPCVD) system [43]. A combined gas flow of Ar, H_2_, and O_2_ was introduced into the LPCVD chamber when the pressure reached 0.01 Pa. The Ar and H_2_ flow rates were kept at 600 sccm and 100 sccm during growth, which was calibrated with flow meters; the O_2_ flow rate varied from 0 to 0.1 sccm in different growth stages. Thermal treatments at 1050 °C to copper foil lasted for 1 h and kept for 2 h for better crystallinity before 0.5 sccm methane was introduced into the LPCVD chamber. In order to obtain graphene with good quality and large size, the growth of graphene lasted for 1 h, and then the system was cooled to RT. Graphene samples were transferred to SiO_2_, NH_2_-SAM/SiO_2_, and F-SAM/SiO_2_ substrates by commonly used method using poly(methyl methacrylate) (PMMA). In order to remove organic residues and contaminants during the transfer process, graphene/SiO_2_, graphene/NH_2_-SAM, and graphene/F-SAM were thoroughly cleaned by acetone vapor. After, all graphene samples were loaded into a quartz tube with base pressure greater than 5 × 10^−2^ Pa, and then heated to 150 °C for 15 min under H_2_ environment.

### 2.3. Characterizations

A multifunctional ultrahigh vacuum (UHV) VT-SPM system (Omicron Instruments, Uppsala, Germany) was used for in situ UPS and X-ray photoelectron spectroscopy (XPS) measurements. The base pressure in the analysis chamber was greater than 3 × 10^−10^ mbar. For UPS measurements, He I (21.2 eV) was the excitation source. A −5 V sample bias was applied during WF measurements. XPS measurements were performed with an Al Kα source. Before deposition, ZnPc and C_60_ were thoroughly degassed overnight, and then thermally evaporated onto graphene, NH_2_-SAM modified graphene, and F-SAM modified graphene in the growth chamber. The deposition rate for ZnPc and C_60_ was 0.1 and 0.2 nm/min, respectively. The nominal thicknesses of ZnPc and C_60_ were determined with the attenuation of Si 2p peak after deposition. Using a Renishaw inVia Raman Microscope, (Renishaw, Gloucestershire, UK) Raman spectra were acquired. The wavelength of the excitation laser was 514 nm. During measurements, focused laser spot of ~1 μm, power of ~1 mW and integration time of 10 s were chosen.

## 3. Results and Discussion

**Figure 1:**
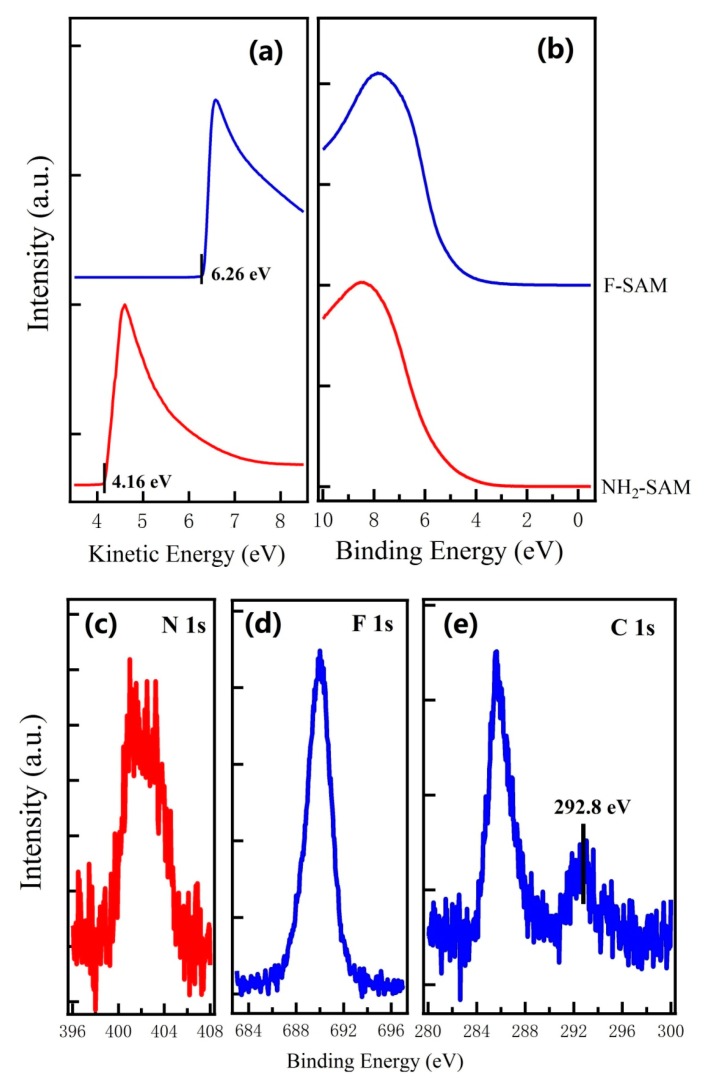
UPS spectra at the (**a**) low kinetic energy region and (**b**) low binding energy region for NH_2_-SAM and F-SAM on SiO_2_ substrates, respectively; XPS spectra of (**c**) N 1s for NH_2_-SAM, and (**d**) F 1s and (**e**) C 1s for F-SAM.

The UPS spectra for NH_2_-SAM and F-SAM on SiO_2_ substrates are shown in Figure 1. The WF of NH_2_-SAM and F-SAM are determined by the UPS spectra at the low kinetic energy region (Figure 1a); the WF of NH_2_-SAM is ~3.46 eV, and it is ~5.56 eV for F-SAM on SiO_2_ substrates, which agrees well with the previously reported values [44]. Figure 1c–e shows the N 1s spectrum for NH_2_-SAM, F 1s and C 1s spectra for F-SAM, respectively. For F-SAM on SiO_2_ substrates, two peaks can be identified in the C 1s region. The peak located at ~292.8 eV derives from carbon atoms in CF_2_ and CF_3_ species; the peak located at ~285.5 eV is attributed to carbon atoms from the alkyl chain of F-SAM. The XPS results shown in Figure 1c–e confirm the successful formation of NH_2_-SAM and F-SAM on SiO_2_ substrates in the present study.

**Figure 2:**
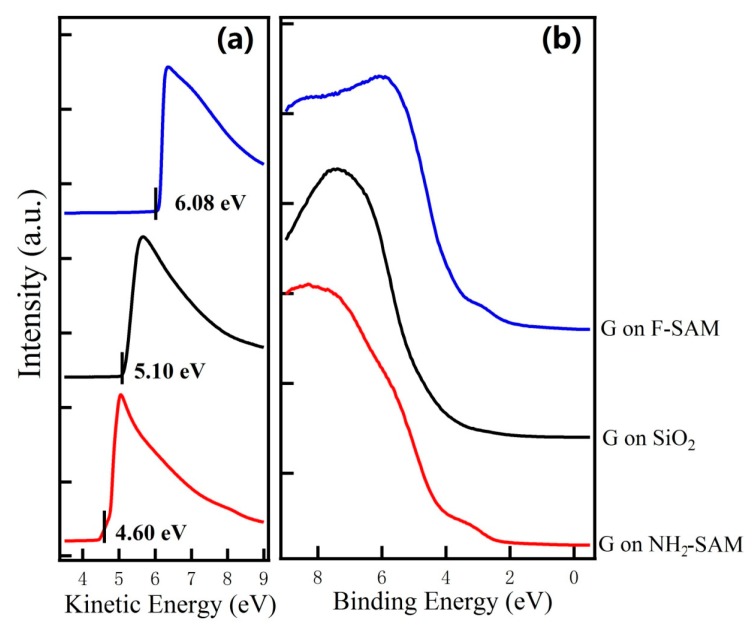
UPS spectra at the (**a**) low kinetic energy region and (**b**) low binding energy region for graphene on NH_2_-SAM, SiO_2_, and F-SAM.

The UPS spectra for graphene on NH_2_-SAM, SiO_2_, and F-SAM are shown in Figure 2. Without surface modification using SAMs, the WF of graphene is ~4.40 eV, showing good consistency with previous reports [45]. In the case of graphene on the NH_2_-SAM, the WF decreases to ~3.90 eV, which is evidenced by the shift of secondary electron cutoff toward the lower kinetic energy part. The decrease of the sample WF suggests that electrons transfer from NH_2_-SAM to graphene and thus it upward shifts its Fermi level. On the other hand, the sample WF change is in the opposite direction for graphene on F-SAM. The secondary electron cutoff shifts to the higher kinetic energy part (Figure 2a), indicating that the WF of graphene on F-SAM increases to ~5.38 eV. Because of the high electron accepting characteristics of the CF_3_ group, electrons transfer from graphene to F-SAM and thus lowers its Fermi level [41]. The manipulation of graphene Fermi level by SAM modification is further confirmed by Raman results (shown in Figure S2). Upward shifts for both G and 2D peak can be observed for graphene on F-SAM, whereas an upward shift for 2D and a downward shift for the G peak is found for graphene on NH_2_-SAM. These shifts of characteristic Raman peaks suggest that doping of graphene has been successfully realized using SAMs [46]. 

**Figure 3:**
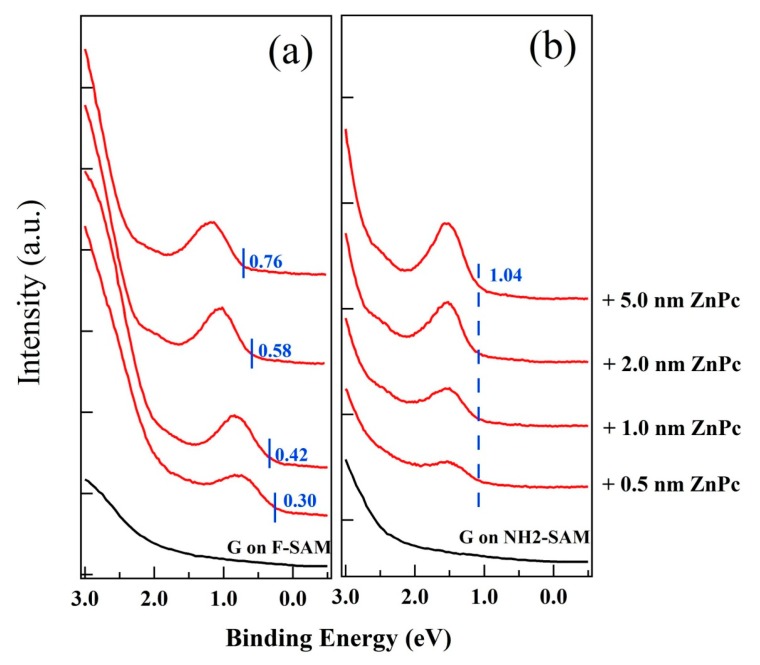
UPS spectra for the growth of ZnPc on (**a**) graphene/F-SAM and (**b**) graphene/ NH_2_-SAM.

**Figure 4:**
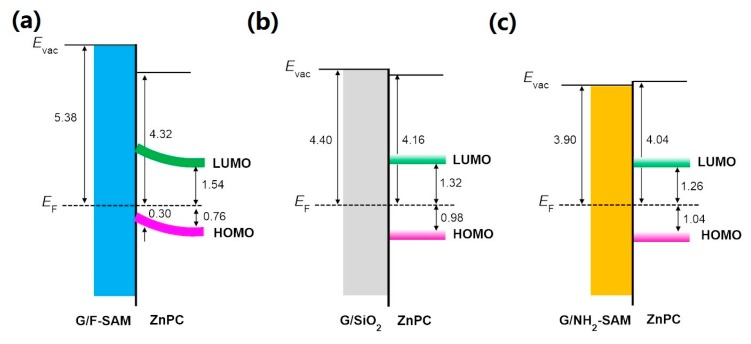
Energy level diagrams of (**a**) ZnPc/graphene on F-SAM, (**b**) ZnPc/graphene, and (**c**) ZnPc/graphene on NH_2_-SAM.

As we mentioned in Section 1, the upper surface of functionalized graphene using SAMs remains intact, and thus no change of molecular orientation and packing structures of subsequently deposited active materials occurs, which helps to separate the impact of the graphene WF from molecular orientation and packing structure changes. In the present study, two widely used organic molecules, ZnPc and C_60_, were chosen to investigate the impact of graphene WF changes. UPS spectra with the increasing thickness of ZnPc up to 5.0 nm on graphene/F-SAM and graphene/NH_2_-SAM are displayed in Figure 3. After the growth of 0.5 nm ZnPc on graphene/F-SAM, ZnPc related emission features begin to emerge, with the highest occupied molecules orbital (HOMO) leading edge located at ~0.30 eV below the substrate Fermi level. After 5.0 nm ZnPc has been deposited, the sample WF decreases from ~5.38 eV to ~4.32 eV (not shown here). We also observe the shift for the HOMO leading edge of ZnPc to the high binding energy part, and it finally locates at ~0.76 eV (Figure 3a), suggesting an upward band bending at the interface. However, the growth of ZnPc on graphene/NH_2_-SAM differs from the growth of ZnPc on graphene/F-SAM, and no notable vacuum level shift is observed with the increasing coverage of ZnPc, which is evidence for vacuum level alignment at the ZnPc/graphene interface on NH_2_-SAM. As shown in Figure 3b, after the growth of 0.5 nm ZnPc on graphene/NH_2_-SAM, we can clearly observe that the HOMO leading edge is at ~1.04 eV. Despite the increasing peak intensity with the increasing coverage, no binding energy shift for the HOMO leading edge of ZnPc is found. After the deposition of 5.0 nm ZnPc, it still locates at ~1.04 eV below the Fermi level. Moreover, similar behavior is observed with the increasing coverage of ZnPc on graphene/SiO_2_ (Figure S2); when the thickness of ZnPc is 5.0 nm, the HOMO leading edge is ~0.98 eV below the Fermi level. Schematic energy level diagrams at the interface between ZnPc and graphene/F-SAM, graphene/SiO_2_, and graphene/NH_2_-SAM are shown in Figure 4. As shown in Figure 2 and Figure S2, the electron accepting characteristics of the F-SAM end group leads to the p-type doping and downward shifts the graphene Fermi level. Therefore, the WF of F-SAM modified graphene increases to ~5.38 eV, which is even higher than the ionization potential of ZnPc (~5.08 eV as shown in Figure 4). If we assume that there is an interfacial vacuum level alignment, the HOMO of ZnPc locates above the graphene Fermi level on F-SAM. In this case, there is no energy barrier for the spontaneous electron transfer from ZnPc to the underlying F-SAM modified graphene. More charge transfer occurs at the interface region compared to that of thicker ZnPc thin films, and hence the upward band bending occurs [47]. With the growth of ZnPc on pristine graphene and NH_2_-SAM modified graphene, the Fermi level locates in the middle of ZnPc band gap, and hence charge transfer is impeded at the interface. As a result, there is little or no shift of vacuum level as well as the HOMO leading edge of ZnPc.

**Figure 5:**
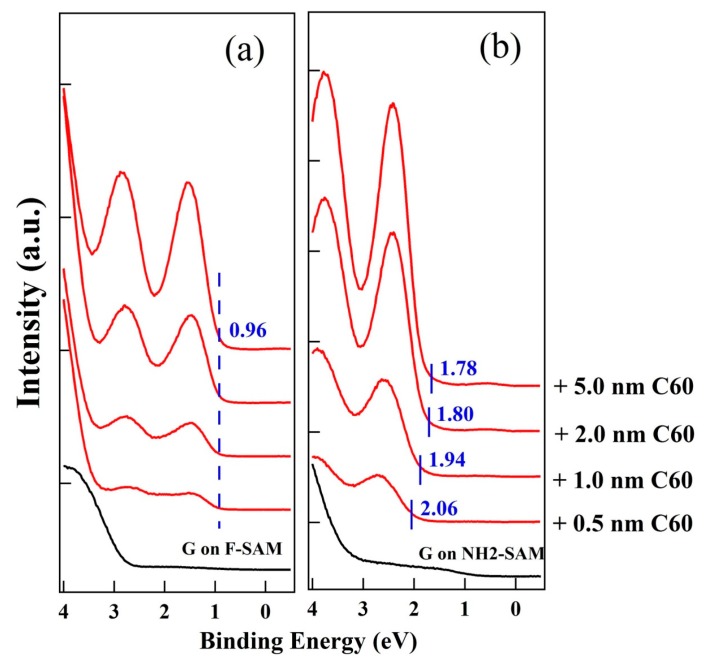
UPS spectra for the growth of C_60_ on (**a**) graphene/F-SAM and (**b**) graphene/NH_2_-SAM.

**Figure 6:**
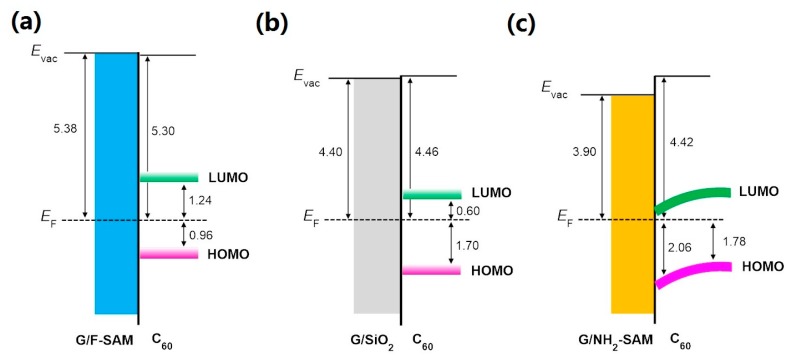
Energy level diagrams of (**a**) C_60_/graphene on F-SAM, (**b**) C_60_/graphene, and (**c**) C_60_/graphene on NH_2_-SAM.

The growth behavior of C_60_, a typical n-type organic semiconductor with relatively high ionization potential [48], on pristine and SAM modified graphene was also investigated in the present study to explore the impact of graphene WF changes. UPS spectra of the growth of C_60_ on graphene/F-SAM and graphene/NH_2_-SAM are shown in Figure 5. On graphene/F-SAM, we do not observe a vacuum level shift during the deposition of C_60_, a clear sign of vacuum level alignment at the interface between C_60_ and F-SAM modified graphene. In addition, no binding energy shift of the C_60_ HOMO leading edge is found. After the deposition of 5.0 nm C_60_, we can see that the HOMO leading edge of C_60_ is ~0.96 eV. The UPS results for the growth of C_60_ on graphene/SiO_2_ are similar to that on graphene/F-SAM. There are no vacuum level and HOMO leading edge shifts during the growth of C_60_ (Figure S4). When the thickness of C_60_ increases to 5.0 nm, it locates at ~1.70 eV below the graphene Fermi level. Unlike the growth of C_60_ on pristine graphene and graphene/F-SAM, an unambiguous downward band bending can be recognized after the growth of C_60_ on graphene/NH_2_-SAM. The HOMO leading edge of C_60_ is ~2.06 eV when 0.5 nm C_60_ has been evaporated onto graphene/NH_2_-SAM, and an upward shift to ~1.78 eV below the Fermi level is observed when the thickness of C_60_ increases to 5.0 nm. Because of the n-type doping of graphene, the WF of NH_2_-SAM modified graphene decreases to ~3.90 eV, which is even lower than the EA of C_60_ (~4.18 eV as shown in Figure 6). Supposing that there is a vacuum level alignment, the energy position of the lowest unoccupied molecular orbital (LUMO) of C_60_ is lower than the graphene Fermi level on NH_2_-SAM. Therefore, spontaneous electron transfer from NH_2_-SAM modified graphene to C_60_ takes place upon deposition, resulting in the downward band bending. The schematic energy level diagrams at the interface between C_60_ and graphene/F-SAM, pristine graphene, and graphene/NH_2_-SAM are shown in Figure 6.

## 4. Conclusions

The impact of graphene WF on interfacial energy levels between graphene and organic molecules has been investigated by tuning its Fermi level using SAMs with different end groups. The WF of graphene on NH_2_-SAM decreases to ~3.90 eV, and increases to ~5.38 eV on F-SAM. Spontaneous electron transfer from ZnPc to F-SAM modified graphene occurs, which is induced by the downward shift of the graphene Fermi level on F-SAM, leading to an upward band bending at the interface. In contrast, we identify a downward band bending at the interface between C_60_ and NH_2_-SAM modified graphene, which can be attributed to the upward shift of the graphene Fermi level on NH_2_-SAM. By using SAMs to manipulate the Fermi level of graphene, the electronic structure at the graphene/organic interface is optimized without disturbing molecular orientation on top, which facilitates the charge injection or collection at the electrode. This can greatly enhance the performance of graphene-based organic electronic devices, and shed light on interfacial engineering approaches for other kinds of transparent electrodes.

## Supplementary Materials

The following are available online at https://www.mdpi.com/2079-4991/9/8/1136/s1, Figure S1: Molecular structures of NH_2_-SAM and F-SAM; Figure S2: Raman spectra for graphene on NH_2_-SAM, SiO_2_, and F-SAM; Figure S3: UPS spectra during the sequential deposition of 5.0 nm ZnPc on graphene; Figure S4: UPS spectra during the sequential deposition of 5.0 nm C_60_ on graphene.
