# Supplementary material for: Impact of Graphene Work Function on the Electronic Structures at the Interface between Graphene and Organic Molecules
Source: Nanomaterials (Basel). 2019 Aug 7;9(8):1136. doi: 10.3390/nano9081136 (PMC6723703; doi:10.3390/nano9081136)
Supplement: Supplementary file 1 [file nanomaterials-09-01136-s001.pdf]

Supplementary material

Impact of graphene work function on the electronic structures at the interface between graphene and organic molecules

HaiTao Wang [1], XiangDong Yang [1], WeiDong Dou [2], Peng Wang [3], QuanLin Ye [1], XuXin Yang [1], BaoXing Li [1,*] and HongYing Mao [1,*]

[1] Department of Physics, Hangzhou Normal University, Hangzhou 310036, China

[2] Laboratory of Low-dimensional Carbon Materials, Physics Department, Shaoxing University, Shaoxing, 312000, China

[3] Department of Applied Physics, College of Electronic and Information Engineering, Shandong University of Science and Technology, Qingdao, 266590, China

* Correspondence: phymaohy@hznu.edu.cn (H.M.) and phybxli@hznu.edu.cn (B.L.);

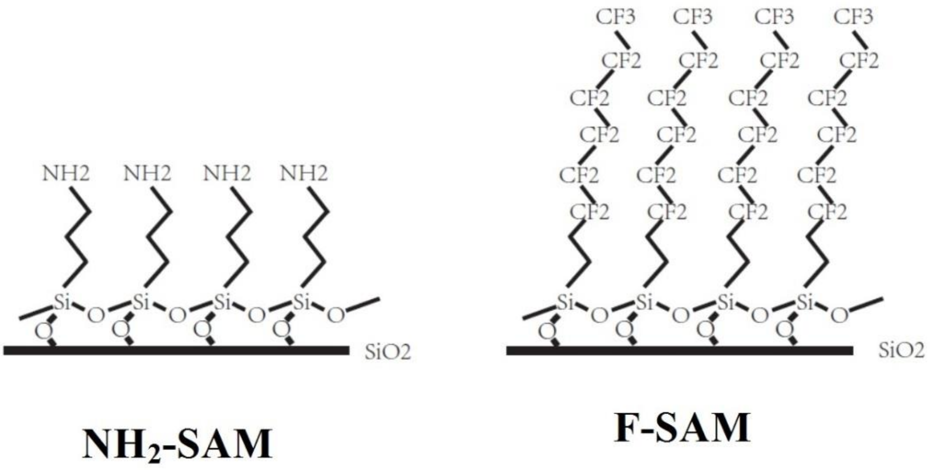

Figure S1 Molecular structures of NH_2-SAM and F-SAM

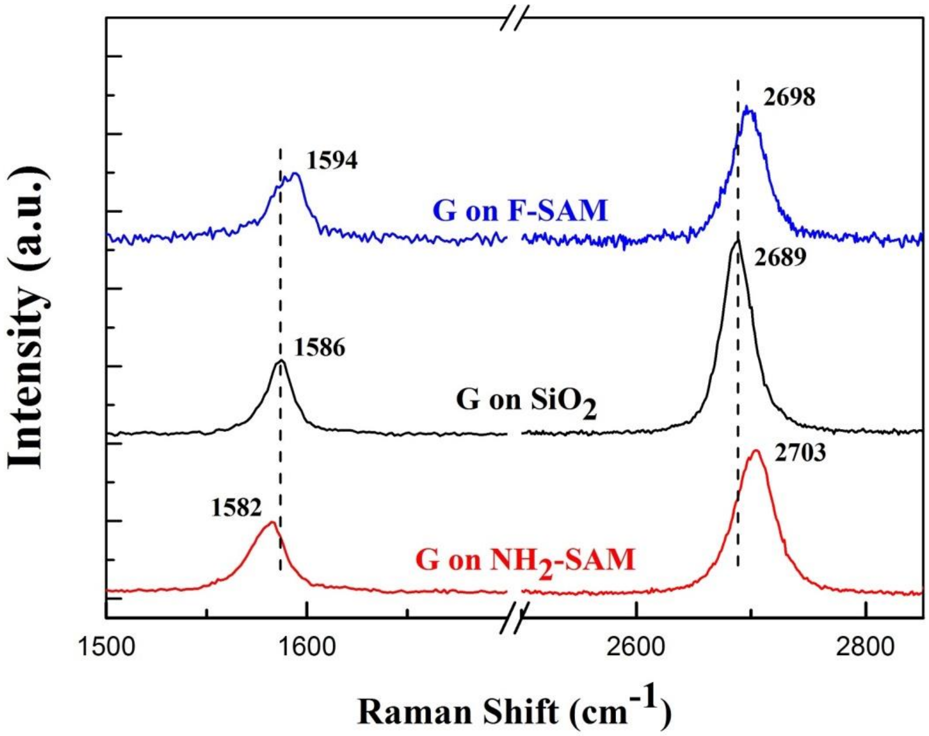

Figure S2. Raman spectra for graphene on NH_2-SAM, SiO_2, and F-SAM, respectively.

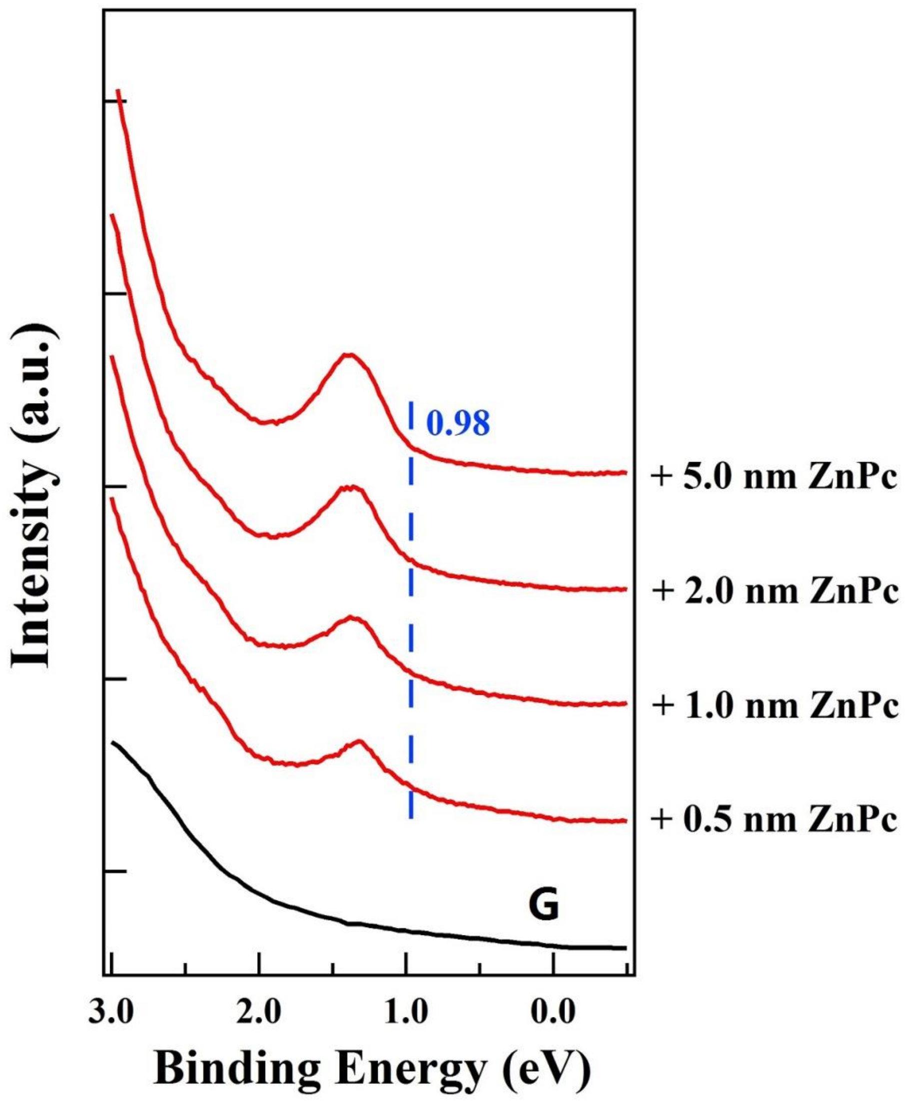

Figure S3. UPS spectra during the sequential deposition of 5.0 nm ZnPc on graphene

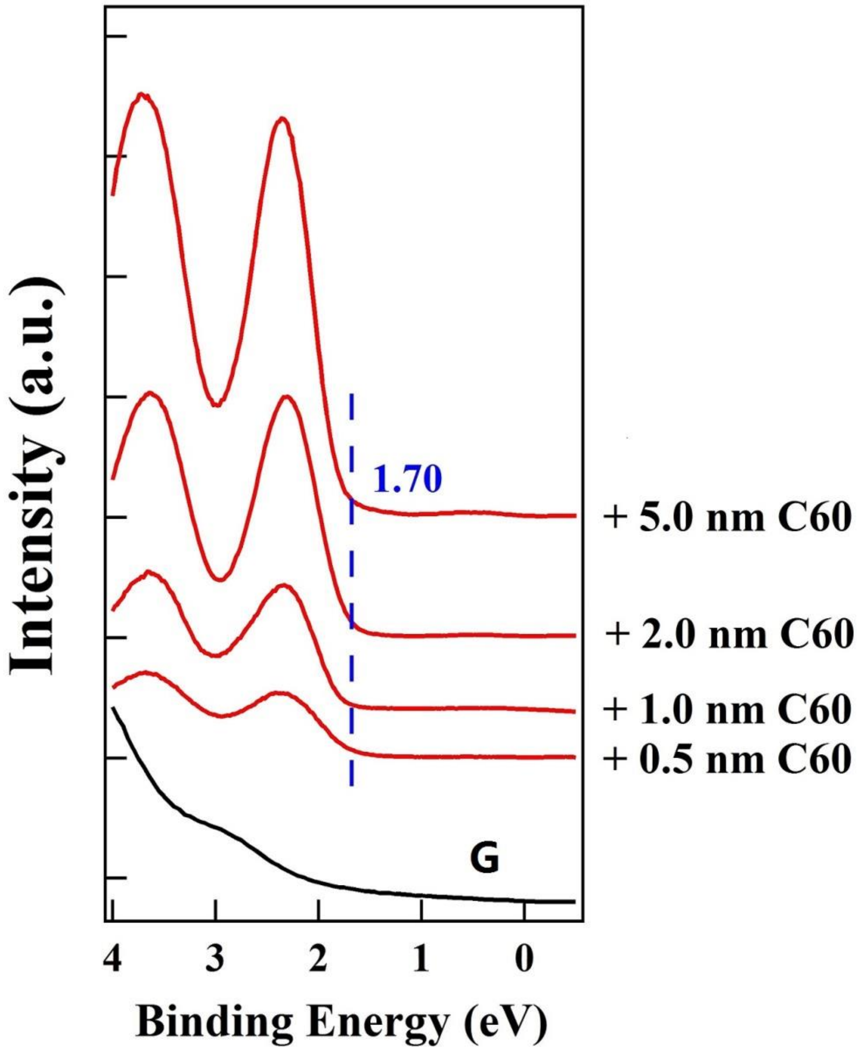

Figure S4. UPS spectra during the sequential deposition of 5.0 nm C_{60} on grapheme.
